# The *Trypanosoma brucei*-Derived Ketoacids, Indole Pyruvate and Hydroxyphenylpyruvate, Induce HO-1 Expression and Suppress Inflammatory Responses in Human Dendritic Cells

**DOI:** 10.3390/antiox11010164

**Published:** 2022-01-15

**Authors:** Hannah K. Fitzgerald, Sinead A. O’Rourke, Eva Desmond, Nuno G. B. Neto, Michael G. Monaghan, Miriam Tosetto, Jayne Doherty, Elizabeth J. Ryan, Glen A. Doherty, Derek P. Nolan, Jean M. Fletcher, Aisling Dunne

**Affiliations:** 1 School of Biochemistry and Immunology, Trinity Biomedical Sciences Institute, Trinity College Dublin, D02 R590 Dublin, Ireland; hfitzge@tcd.ie (H.K.F.); siorourk@tcd.ie (S.A.O.); evadesmond@gmail.com (E.D.); DENOLAN@tcd.ie (D.P.N.); Fletchj@tcd.ie (J.M.F.); 2 Trinity Centre for Biomedical Engineering, Trinity Biomedical Sciences Institute, Trinity College Dublin, D02 R590 Dublin, Ireland; neton@tcd.ie (N.G.B.N.); monaghmi@tcd.ie (M.G.M.); 3 Centre for Colorectal Disease, St. Vincent’s University Hospital, School of Medicine, University College Dublin, D04 YN26 Dublin, Ireland; mtosetto@ucd.ie (M.T.); Jayne.Doherty@svph.ie (J.D.); gdoherty@svhg.ie (G.A.D.); 4 Department of Biological Sciences, Health Research Institute, University of Limerick, V94 T9PX Limerick, Ireland; Elizabeth.Ryan@ul.ie; 5 School of Medicine, Trinity Biomedical Sciences Institute, Trinity College Dublin, D02 R590 Dublin, Ireland

**Keywords:** heme oxygenase 1, *Trypanosoma brucei*, inflammatory bowel disease, aromatic ketoacids, dendritic cells, immunomodulation, anti-inflammatory therapies

## Abstract

The extracellular parasite and causative agent of African sleeping sickness *Trypanosoma brucei* (*T. brucei*) has evolved a number of strategies to avoid immune detection in the host. One recently described mechanism involves the conversion of host-derived amino acids to aromatic ketoacids, which are detected at relatively high concentrations in the bloodstream of infected individuals. These ketoacids have been shown to directly suppress inflammatory responses in murine immune cells, as well as acting as potent inducers of the stress response enzyme, heme oxygenase 1 (HO-1), which has proven anti-inflammatory properties. The aim of this study was to investigate the immunomodulatory properties of the *T. brucei*-derived ketoacids in primary human immune cells and further examine their potential as a therapy for inflammatory diseases. We report that the *T. brucei*-derived ketoacids, indole pyruvate (IP) and hydroxyphenylpyruvate (HPP), induce HO-1 expression through Nrf2 activation in human dendritic cells (DC). They also limit DC maturation and suppress the production of pro-inflammatory cytokines, which, in turn, leads to a reduced capacity to differentiate adaptive CD4^+^ T cells. Furthermore, the ketoacids are capable of modulating DC cellular metabolism and suppressing the inflammatory profile of cells isolated from patients with inflammatory bowel disease. This study therefore not only provides further evidence of the immune-evasion mechanisms employed by *T. brucei*, but also supports further exploration of this new class of HO-1 inducers as potential therapeutics for the treatment of inflammatory conditions.

## 1. Introduction

Infection of the mammalian vasculature and central nervous system (CNS) with the extracellular protozoan parasite *Trypanosoma brucei* (*T. brucei*) can lead to fatal human sleeping sickness, also known as African trypanosomiasis. Like most parasites, trypanosomes are continuously challenged by the host-immune system, however, they have evolved very effective evasion strategies in order to maintain infection and prolong the host’s survival [1]. An infection with *T. brucei* is accompanied by the excretion of high levels of ketoacids into the host’s bloodstream [2,3], a phenomenon that was, until recently, largely unexplained. The ketoacids are derived from the conversion of aromatic amino acids (tryptophan, tyrosine, and phenylalanine) to indole pyruvate (IP), hydroxyphenylpyruvate (HPP), and phenyl pyruvate (PP), through the action of a cytoplasmic aspartate aminotransferase (TbcASAT), which is expressed by the parasite.

In recent years, we, and others, have demonstrated that these molecules have potent immunomodulatory properties, which likely contribute to the suppression of host immune responses, but that may also be exploited for potential therapeutic benefit [4,5,6,7,8]. For example, IP and HPP are potent inducers of the immunosuppressive enzyme heme oxygenase-1 (HO-1) in murine glia and macrophages [5]. This occurs in a nuclear factor (erythroid-derived 2)-like 2 (Nrf2) dependent manner and leads to a reduction in LPS-induced pro-inflammatory cytokines and innate immune cell maturation. Indeed, HO-1 has well established anti-inflammatory properties and is induced by a vast number of stimuli during oxidative stress and inflammation. It catalyses the conversion of heme to biliverdin with the liberation of free iron and carbon monoxide (CO). Biliverdin is then converted to bilirubin by biliverdin reductase [9,10] and both biliverdin and bilirubin are considered powerful antioxidants, while many of the anti-inflammatory effects of HO-1 are attributed to CO. The potent anti-inflammatory properties of HO-1 are underlined by rare HO-deficiencies in humans and HO-1 knockout mice, which, along with the expected sensitivity to oxidative stress, show high levels of chronic inflammation [11,12,13]. Unsurprisingly, induction of HO-1 with known and novel HO-1 inducers is now being explored as a therapy for a number of autoimmune and inflammatory diseases and we have recently reviewed this in detail elsewhere [14].

In addition to HO-1 induction, *T. brucei*-derived ketoacids are also capable of inhibiting HIF-1α–induced pro-IL-1β expression, as well as prostaglandin production, an effect which was shown to be dependent on the activation of the aryl hydrocarbon receptor [4,5,8]. The immunomodulatory effects of IP and HPP have also been confirmed in animal models of disease. In a murine model of skin damage caused by exposure to ultraviolet B radiation, administration of IP resulted in a reduction in damage lesions and expression of the pro-inflammatory cytokines, IL-1β and IL-6 [6]. Furthermore, IP administration reduced disease severity in the DSS colitis model, and this was accompanied by a decrease in the expression of pro-inflammatory cytokines IL-12, TNF, IFNγ, and IL-1β, and an increase in the expression of the anti-inflammatory cytokine IL-10 [7]. A reduction in Th1 cells, as well as a reduced capacity for dendritic cells (DC) to activate T cells, was also observed [7].

Despite the recent interest in these ketoacids as immunomodulators, to date, they have been primarily studied in murine immune cells with very little evidence to support their mechanism of action in human immune cells. In this study, we investigate the immunomodulatory properties of the *T. brucei*-derived ketoacids, IP and HPP, in primary human immune cells and further examine their potential as a therapy for inflammatory diseases.

## 2. Materials and Methods

### 2.1. Reagents and Chemicals

4-hydroxyphenylpyruvic acid (HPP) and indole-3-pyruvic acid (IP) were purchased from Merck (Darmstadt, Germany) and dissolved in RPMI to a final concentration of 2 mM before use. Ultrapure lipopolysaccharide (LPS) from *E. Coli* O111:B4 was purchased from Enzo Life Sciences (Bruxelles, Belgium). Complete RPMI or complete IMDM were prepared by supplementing with 10% foetal bovine serum (FBS), 2 mM L-glutamine, 100 U/mL penicillin, and 100 µg/mL streptomycin, which were all purchased from Merck (Darmstadt, Germany). Lymphoprep is manufactured by Axis-Shield poC (Dundee, Scotland). GM-CSF and IL-4 were purchased from Miltenyi Biotec (Bergisch Gladbach, Germany). The protease inhibitor cocktail and High-Pure RNA Isolation Kit were purchased from Roche (Basel, Switzerland). The western blot antibodies for Nrf2, HK2, p-AMPK, t-AMPK, p62 and LC3 were all purchased from Cell Signalling Technology (Danvers, MA, USA), while the antibody for HO-1 was purchased from Enzo Life Sciences (Bruxelles, Belgium), and the β-actin antibody and secondary anti-rabbit were purchased from Merck (Darmstadt, Germany). The Fixable Viability Dye, antibodies for CD40, CD80, CD83, and CD86, and Annexin V & PI staining kit were all purchased from eBioscience (San Diego, CA, USA). The DQ-Ovalbumin, CD3 and IL-17 flow antibodies, FIX & PERM™ Cell Permeabilization Kit, anti-CD3, and all ELISA kits used were purchased from Invitrogen (Waltham, MA, USA). The High-Capacity cDNA reverse transcription kit was purchased from Applied Biosystems (Beverly, MA, USA) and the iTaq Universal SYBR Green mastermix from Bio-Rad (Hercules, CA, USA). The MagniSort Human CD14 Positive Selection kit and MagniSort Human CD4 T cell Positive Selection kit were purchased from Thermo Fisher Scientific (Waltham, MA, USA). The Zombie NIR^TM^ Fixable Viability kit was purchased from BioLegend (San Diego, CA, USA). The antibodies for CD8, ki67 and IFNγ were purchased from BD biosciences (East Rutherford, NJ, USA). The Complete XF assay medium was purchased from Agilent (Santa Clara, CA, USA). The Corning™ Cell-Tak Cell and Tissue Adhesive was purchased from Fisher Scientific (Waltham, MA, USA). Oligomycin was purchased from Cayman Chemicals (Ann Arbor, MI, USA) and carbonyl cyanide-p trifluoromethoxyphenylhydrazone (FCCP) from Santa Cruz biotechnology (Dallas, TX, USA). The HEK-Blue^TM^ hTLR4 assay system was purchased from InvivoGen (San Diego, CA, USA). Dulbecco’s phosphate buffered saline (PBS), enhanced chemiluminescent substrate, phosphatase inhibitor cocktail, Antioxidant assay kit, Phorbol 12-myristate 13-acetate (PMA), ionomycin, brefeldin A, rotenone, antimycin A, and 2-deoxy-D-glucose (2-DG) were all purchased from Merck (Darmstadt, Germany).

### 2.2. Human Blood Samples

The Irish Blood Transfusion Service (IBTS) at St. James’s Hospital in Dublin supplied leukocyte-enriched buffy coats for these studies, from donors who provided informed written consent. Ethical approval was obtained from the research ethics committee of the School of Biochemistry and Immunology at Trinity College Dublin, and all experiments were carried out in accordance with the Declaration of Helsinki. Peripheral blood mononuclear cells (PBMC) were isolated by a density gradient centrifugation. The cells were cultured in complete RPMI medium and maintained in humidified incubators at 37 °C with 5% CO_2_.

### 2.3. Dendritic Cell Culture

The MagniSort Human CD14 Positive Selection kit was used according to the manufacturer’s protocol to positively select for CD14^+^ monocytes from PBMC. CD14^+^ monocytes were then cultured at 1 × 10^6^ cells/mL in complete RPMI and supplemented with GM-CSF (50 ng/mL) and IL-4 (40 ng/mL) to generate monocyte-derived DC. On day three of culture, half of the media was replaced with fresh media supplemented with cytokines at the same starting concentration. On day six, non-adherent and loosely adherent cells were gently removed for use. The purity of CD14^lo^DC-SIGN^+^ DC was confirmed by flow cytometry and was routinely >95%. DC were cultured at 1 × 10^6^ cells/mL for all further assays.

### 2.4. Western Blot Experiments

For detection of HO-1 expression, DC were cultured in the presence of HPP or IP (250–1000 µM) for 3, 6, and 24 h, or treated with the Nrf2 inhibitor ML385 (10 µM) for 1 h prior to treatment with HPP or IP at 1000 µM for 24 h. Cell lysates were prepared by washing cells in PBS prior to lysis in RIPA buffer (Tris 50 mM; NaCl 150 mM; SDS 0.1%; Na.Deoxycholate 0.5%; Triton X 100). For detection of Nrf2 expression, DC were cultured in the presence of HPP (1000 µM) or IP (1000 µM) for 6 or 24 h, then washed in PBS and lysed in Laemmli loading buffer. For detection of hexokinase 2 (HK2) expression, DC were cultured in the presence of HPP or IP at 1000 µM for 6 h prior to stimulation with LPS (100 ng/mL) for 12 h, then washed in PBS and lysed in Laemmli loading buffer. For detection of p-AMPK and t-AMPK expression, DC were cultured in the presence of HPP or IP (both 1000 µM) for 15 min, then washed in PBS and lysed in Laemmli loading buffer. For the detection of p62 and LC3, DC were cultured in the presence of HPP or IP (both 1000 µM) for 6, 12, or 24 h, then washed in PBS, lysed in Laemmli loading buffer, and sonicated. All samples were lysed in the presence of a protease inhibitor cocktail and phosphatase inhibitor cocktail set. Samples were electrophoresed and transferred to PVDF. The membranes were incubated overnight at 4 °C with monoclonal antibodies specific for HO-1, Nrf2, HK2, p-AMPK, t-AMPK, p62, and LC3. The membranes were washed in TBS–Tween prior to incubation with an anti-rabbit streptavidin-conjugated secondary antibody for 2 h at room temperature. A Bio-Rad ChemiDoc MP system was used for developing the blots. The membranes were subsequently re-probed with a loading control, β-actin, in order to normalise the protein of interest to the loading control for densitometric analysis.

### 2.5. Antioxidant Assay

DC were cultured in the presence of IP or HPP (both 1000 µM) for 1 h. The cells were lysed by sonication in ice-cold PBS and centrifuged at 14,000 rpm for 10 min to pellet any debris. The total antioxidant capacity of the cells was analysed using an Antioxidant assay kit according to the manufacturer’s protocol. The assay measures the reduction of Cu^2+^ by an antioxidant to Cu^+^, which can subsequently form a coloured complex with a dye reagent in the kit. The absorbances of the samples were read at 570 nm and compared to the absorbances of a range of known concentrations of Trolox standards. The data is displayed as the total antioxidant capacity of the cells expressed as an equivalent concentration of Trolox (µM).

### 2.6. DC Flow Cytometry Experiments

DC were collected upon completion of the experiment, washed in PBS, and stained accordingly. DC were stained using an Annexin V & PI staining kit according to the manufacturer’s protocol for viability assays. For the maturation marker assay, DC were initially stained with Fixable Viability Dye, for dead cells, and subsequently with fluorochrome-conjugated antibodies for CD40, CD80, CD83, and CD86. In order to measure the phagocytic capacity, DC were incubated with RPMI-containing DQ-Ovalbumin (500 ng/mL) for 20 min at 37 °C, before transferring to 4 °C for a further 10 min incubation to stop the uptake of the model antigen. DC were then washed in PBS and immediately acquired. All of the above experiments were acquired on a BD FACS Canto II, and the analysis was performed using FlowJo v.10 software (Tree Star Inc., Ashland, OR, USA).

### 2.7. Quantitative Real-Time PCR

DC were cultured in the presence of HPP or IP (both 1000 µM) for 6 or 24 h. Detection of NAD(P)H dehydrogenase (quinone 1) (NQO1, accession number P15559) expression and glutathione reductase (GSR, accession number P00390) expression were measured using quantitative real-time PCR. RNA was first extracted using the High-Pure RNA Isolation Kit, followed by cDNA synthesis using the High-Capacity cDNA reverse transcription kit. iTaq Universal SYBR Green mastermix was used in the reaction along with relevant primers—the sequences are listed in Table 1. A Bio-Rad CFX96 Real-Time System was used to carry out the reaction. mRNA expression levels for genes of interest were quantified and normalized to the housekeeping (β-actin) mRNA levels.

### 2.8. DC ELISA Experiments

For detection of cytokines, DC were cultured in the presence of HPP or IP (500–1000 µM) for 6 h prior to stimulation with LPS (100 ng/mL) for 24 h. Concentrations of IL-12p70, IL-23, TNF, IL-6, and IL-10 were quantified from supernatants by ELISA as per the manufacturers’ protocols.

### 2.9. DC-CD4^+^ T Cell Co-Cultures

The MagniSort Human CD4 T cell Positive Selection kit was used according to the manufacturer’s protocol to isolate CD4^+^ T cells from PBMC. CD4^+^ T cells were co-cultured with allogeneic DC that had been pre-treated with IP or HPP and stimulated with LPS as before. The cells were co-cultured at a ratio of 10:1 T cells to DC for five days with no ketoacid present. The supernatants were removed for analysis of IL-10 by ELISA prior to restimulation of the cells in complete RPMI in the presence of 50 ng/mL PMA, 500 ng/mL ionomycin, and 5 µg/mL brefeldin A for 4 h. The cells were washed in PBS and stained for viability (Zombie NIR^TM^ Fixable Viability kit) for 15 min at room temperature. The cells were then washed again in PBS and stained with fluorochrome-conjugated antibodies for surface markers CD3 and CD8 for 15 min at room temperature. The cells were then washed and fixed (FIX & PERM™ Cell Permeabilization Kit) for 15 min at room temperature. Finally, the cells were washed and stained for intracellular markers Ki67 and IFNγ in permeabilization buffer for 15 min at room temperature. A BD LSRFortessa flow cytometer was used to acquire samples, and analysis was performed using FlowJo v.10 software.

### 2.10. Two-Photon Fluorescence Lifetime Imaging Microscopy (FLIM)

DC were cultured in the presence of HPP (1000 µM) for 6 h prior to stimulation with LPS (100 ng/mL) for 12 h. Two-photon excited NAD(P)H- Fluorescence Lifetime Imaging Microscopy (FLIM) was used to measure the levels of free and protein-bound NADH within these cells, and was performed on a custom multiphoton system (further details regarding experimental setup can be found at the following [15,16]). At least three images for each model were acquired. Afterwards, regions of interest (ROI) were selected, and the NAD(P)H fluorescence decay was analysed.

For the NAD(P)H fluorescence decay analysis, an overall decay curve was generated by the contribution of all pixels in the ROI area. Afterwards, it was fitted with a double exponential decay curve (Equation (1)):

(1) $$\;I\left(t\right)=\alpha_{1}e^{-\frac{t}{\tau_{1}}}+\alpha_{2}e^{-\frac{t}{\tau_{2}}}+c$$

*I*(*t*) represents the fluorescence intensity at time (*t*) after laser excitation. *α*_1_ and *α*_2_ represent the fraction of the overall signal comprised of a short and long lifetime component, respectively. *τ_1_* and *τ_2_* are the long and short lifetime components, respectively. *C* corresponds to background light. X^2^ is calculated to evaluate the goodness of multi-exponential fit to the raw fluorescence decay data—the lowest χ^2^ values were considered in this study.

For NAD(P)H, a two-component fit was used to differentiate between the free (*τ*_1_) and protein-bound (*τ*_2_) NAD(P)H. The average lifetime (*τ_avg_*) of NAD(P)H for each pixel is calculated by a weighted average of both the free and bound lifetime contributions (Equation (2)):

(2) $$\tau_{avg}=\frac{\left(\alpha_{1}\times \tau_{1}\right)+\left(\alpha_{2}\times \tau_{2}\right)}{\left(\alpha_{1}+\alpha_{2}\right)}$$

### 2.11. Metabolic Profiling Using Seahorse Analysis

DC were cultured in the presence of IP or HPP (both 1000 µM) for 6 h prior to stimulation with LPS (100 ng/mL) for 12 h. The cell culture medium was replaced with complete XF assay medium (pH of 7.4, supplemented with 10 mM glucose, 1 mM sodium pyruvate, 2 mM L-glutamine) and DC were then transferred at a density of 2 × 10^5^ cells/well to a Seahorse 96-well microplate, which was coated with Corning™ Cell-Tak Cell and Tissue Adhesive and incubated in a non-CO_2_ incubator. Blank wells were prepared containing XF assay medium only to subtract the background oxygen consumption rate (OCR) and extracellular acidification rate (ECAR) during analysis. Oligomycin (1 mM), FCCP (1 mM), rotenone (500 nM), antimycin A (500 nM), and 2-DG (25 mM) were prepared in XF assay medium. Inhibitors were loaded into the appropriate injection ports on the cartridge plate and incubated for 10 min in a non-CO_2_ incubator at 37 °C. Oligomycin, FCCP, rotenone and antimycin A, and 2-DG were sequentially injected while the OCR and ECAR readings were simultaneously measured. Wave software (Agilent Technologies, Santa Clara, CA, USA) was used to analyse the results. The rates of basal glycolysis, max glycolysis, glycolytic reserve, basal respiration, max respiration, and respiratory reserve were calculated as detailed in the manufacturer’s protocol and as supplied in Supplementary Table S1.

### 2.12. IBD Patient PBMC Experiments

This study received ethical approval from St Vincent’s University Hospital Ethics and Medical Research Committee to take blood samples from consenting patients (*N* = 14) attending a specialist outpatient clinic for inflammatory bowel disease (IBD). PBMC were isolated as above and frozen at −80 °C. PBMC were thawed and treated with either IP or HPP (250–1000 μM) for 6 h prior to stimulation with anti-CD3 (1 μg/mL) for 12 h. The media was then removed and replaced with fresh media to circumvent issues that occur as the compounds become fluorescent after incubations over long periods of time, and cells were maintained in the presence of anti-CD3 for a further four days. The supernatants were then removed for analysis of IL-10, IFNγ, and IL-17A by ELISA. PBMC were restimulated in complete IMDM medium in the presence of 50 ng/mL PMA, 500 ng/mL ionomycin, and 5 µg/mL brefeldin A for 4 h. The cells were washed in PBS and stained for viability (Zombie NIR^TM^ Fixable Viability kit) for 15 min at room temperature. The cells were then washed in PBS and stained with fluorochrome-conjugated antibodies for surface markers CD3 and CD8 for 15 min at room temperature. After this, the cells were then washed and fixed (FIX & PERM™ Cell Permeabilization Kit) for 15 min at room temperature. Finally, the cells were washed and stained for intracellular markers Ki67, IFNγ and IL-17 in permeabilization buffer for 15 min at room temperature. The cells were then washed and acquired on a BD LSRFortessa flow cytometer. The analysis was performed with FlowJo v.10. All antibodies used in this experiment were chosen carefully to avoid channels which still had some fluorescence issues, despite the steps taken to overcome this.

### 2.13. Assessment of Endotoxin Contamination

The HEK-Blue^TM^ hTLR4 assay system was used to test IP and HPP for LPS contamination. HEK-blue cells (5 × 10^5^ cells/mL) expressing TLR4 were stimulated with LPS (0.1–100 ng/mL; positive control), or HPP or IP (both 1000 µM) for 24 h. Supernatants from the HEK-blue cells were incubated with HEK-blue detection medium, to measure SEAP expression, for 30 min at 37 °C and absorbance was read at 650 nm.

### 2.14. Statistical Analysis

Prism 9 software (GraphPad Software Inc., San Diego, CA, USA) was used to perform the statistical analysis on all datasets. A repeated measures one-way ANOVA, with either Dunnett’s or Šídák’s post hoc test, as appropriate, was used for analysis of three or more datasets. A Paired Student’s *t*-test was used for the analysis of only two datasets. The analysis of datasets with more than one variable were performed using a two-way ANOVA with Šídák’s multiple comparisons post hoc test. Asterisks are used in the figures to denote *p* values < 0.05, which were considered significant.

## 3. Results

### 3.1. HPP and IP Upregulate HO-1 in Primary Human DC

**Figure 1:**
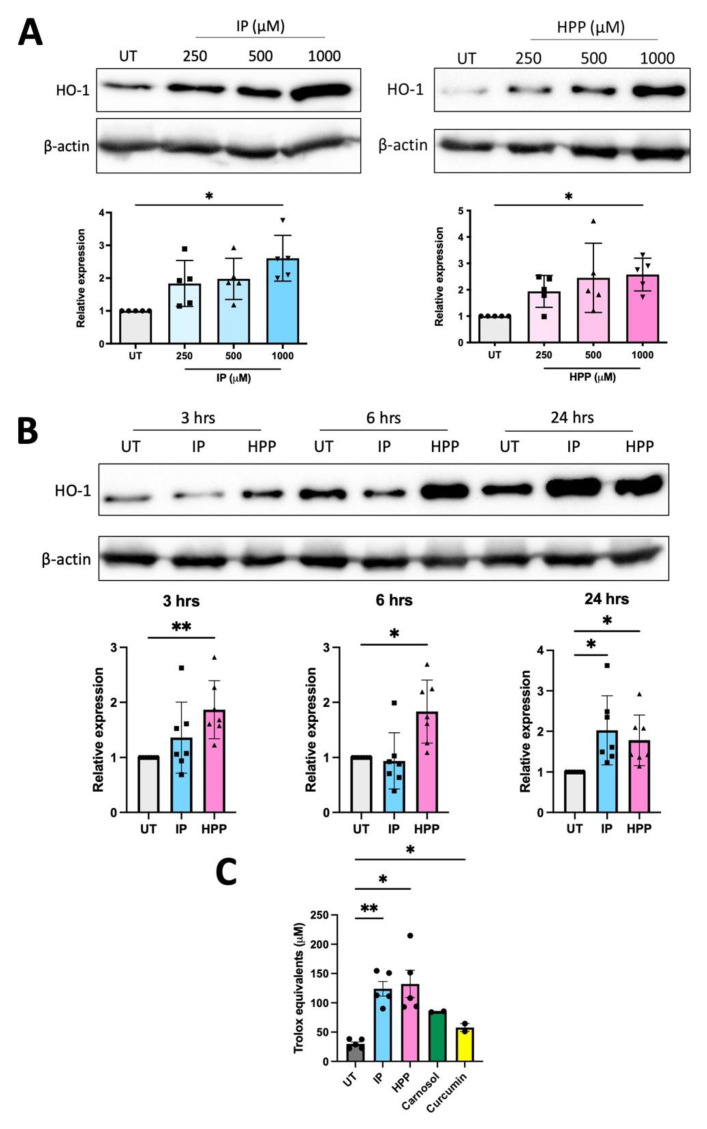
Hydroxyphenylpyruvate (HPP) and indole pyruvate (IP) upregulate HO-1 in primary human dendritic cells (DC). (**A**) Primary human DC were left untreated (UT) or incubated with HPP or IP (250–1000 μM) for 24 h. HO-1 expression was detected by western blot. Densitometry results shown are mean ± SEM of the relative expression of HO-1: β-actin from five healthy donors. (**B**) DC were left UT or incubated with HPP or IP at 1000 μM for 3, 6, or 24 h. HO-1 expression was detected by western blot. Densitometry results shown are mean ± SEM of the relative expression of HO-1: β-actin from seven healthy donors. (**C**) Primary human DC were left UT or incubated with HPP or IP at 1000 μM, or carnosol or curcumin (both 10 μM), for 1 h. Total antioxidant capacity of the cells was determined and expressed as an equivalent concentration of Trolox (μM). Pooled data showing the mean (±SEM) from five healthy donors. Repeated measures one-way ANOVA, with Dunnett’s multiple comparisons post hoc test, was used to determine statistical significance by comparing means of treatment groups against the mean of the control group (** *p* < 0.01, * *p* < 0.05). ImageLab (Bio-Rad) software was used to perform densitometric analysis.

We have previously shown that HPP and IP are capable of inducing HO-1, as well as having immunomodulatory effects, in murine macrophages and glia [5]. Therefore, we sought to investigate if *T. brucei*-derived ketoacids have similar effects in primary human DC, as these are crucial immune cells linking the innate and adaptive immune system. DC were treated with HPP or IP (500–1000 μM) for 24 h (these concentrations are similar to the levels of aromatic ketoacids in circulation close to the peak of parasitaemia during trypanosomiasis and have been used in previously published studies [4,5,8,17]). Both HPP and IP were found to be non-toxic to human DC, having no effect on cell viability at these concentrations, and were confirmed to be endotoxin free (Supplementary Figures S1 and S2). HO-1 expression in HPP- and IP-treated DC was next examined by western blot at a range of timepoints (3, 6, and 24 h) and concentrations (250–1000 μM). Immature DC constitutively expressed HO-1 in line with previous reports [18,19,20], however, treatment with either HPP or IP resulted in a trend towards increased expression of HO-1 at all concentrations tested with a significance observed at 1000 μM in each case (Figure 1A). At this concentration, significant upregulation of HO-1 occurred within 3 h of HPP treatment, while significant induction of HO-1 occurred following 24 h treatment with IP (Figure 1B). Both IP and HPP also increased the total antioxidant capacity of DC and this was observed within 1 h of incubation with either compound (Figure 1C). The ketoacids also appear to be more potent antioxidants than the established HO-1 inducers, carnosol and curcumin (Figure 1C). These results indicate that IP and HPP rapidly enhance the total antioxidant capacity of human DC, while also upregulating the stress-response protein, HO-1.

### 3.2. HPP and IP Induce HO-1 through Nrf2 Activation

**Figure 2:**
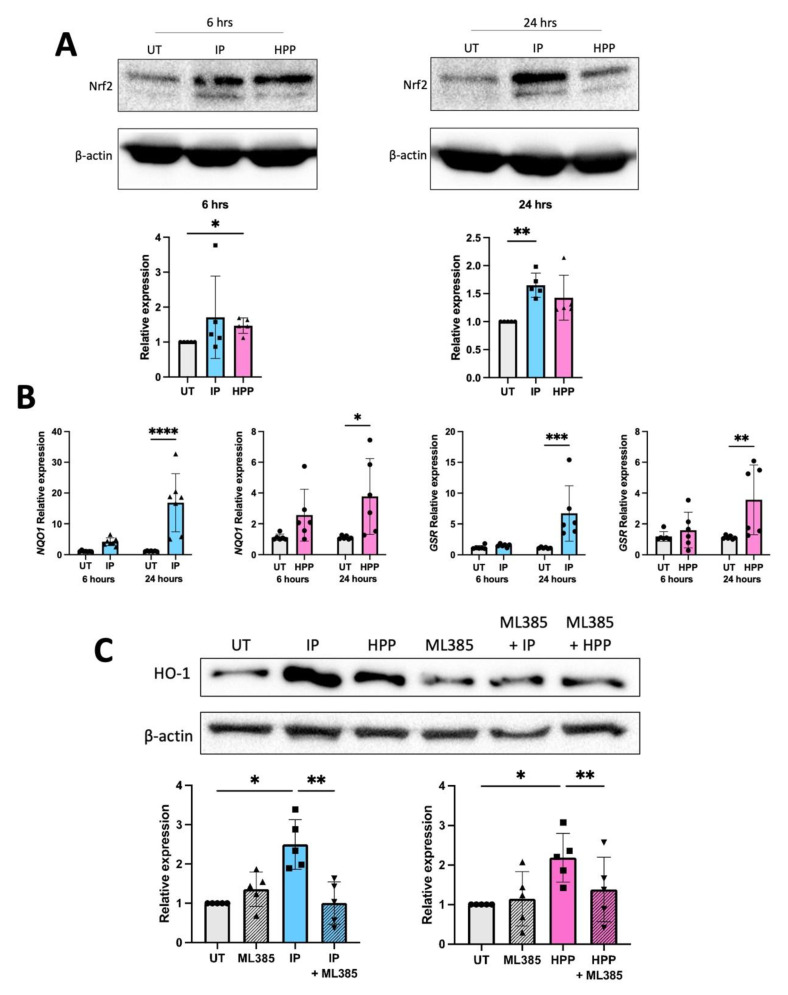
HPP and IP induce HO-1 through Nrf2 activation. (**A**) Primary human DC were left untreated (UT) or incubated with HPP or IP at 1000 μM for 6 or 24 h. Nrf2 expression was measured by western blot. Densitometry results shown are mean ± SEM of the relative expression of Nrf2: β-actin from five healthy donors. (**B**) Primary human DC were left UT or incubated with HPP or IP at 1000 µM for 24 h. mRNA expression of the Nrf2-dependent genes, NQO-1 and GSR, were measured by RT-PCR. Results show mean (±SEM) for six healthy donors. (**C**) Primary human DC were pre-treated either with or without the Nrf2 inhibitor ML385 (10 μM) for 1 h, prior to incubation with HPP or IP at 1000 μM for 24 h. HO-1 expression was measured by western blot. Densitometry results shown are mean ± SEM of the relative expression of HO-1: β-actin from five healthy donors. (**A**) One-way ANOVA, with Dunnett’s multiple comparisons post hoc test, was used to determine statistical significance. (**B**) Two-way ANOVA, with Šídák’s multiple comparisons post hoc test, was used to determine statistical significance. (**C**) One-way ANOVA, with Šídák’s multiple comparisons post hoc test, was used to determine statistical significance (**** *p* < 0.0001, *** *p* < 0.001, ** *p* < 0.01, * *p* < 0.05). ImageLab (Bio-Rad) software was used to perform densitometric analysis.

We have previously reported that HPP and IP activate Nrf2 in murine immune cells, and this is likely the mechanism through which they upregulate HO-1 [5]. We therefore sought to investigate if they have a similar mechanism of action in human DC. To test this, DC were treated with HPP or IP (1000 μM) for 6 and 24 h, and Nrf2 protein expression was measured by western blot. An increase in Nrf2 protein was observed in HPP-treated DC at 6 h (Figure 2A), while IP-treated DC showed increased Nrf2 protein expression (and therefore accumulation) at 24 h (Figure 2A). Both HPP and IP were also found to drive mRNA expression of the additional Nrf2-regulated genes, NQO1 and GSR, further confirming their ability to activate this transcription factor (Figure 2B). Finally, treatment of DC with the Nrf2 inhibitor ML385 (10 μM, a non-toxic dose, Supplementary Figure S3) for 1 h, prior to treatment with either HPP or IP (1000 μM) for 24 h, resulted in a significant decrease in the expression of HO-1 (Figure 2C), further confirming that ketoacid driven HO-1 expression is regulated by Nrf2.

### 3.3. HPP and IP Reduce the Production of Pro-Inflammatory Cytokines in LPS-Stimulated Human DC

**Figure 3:**
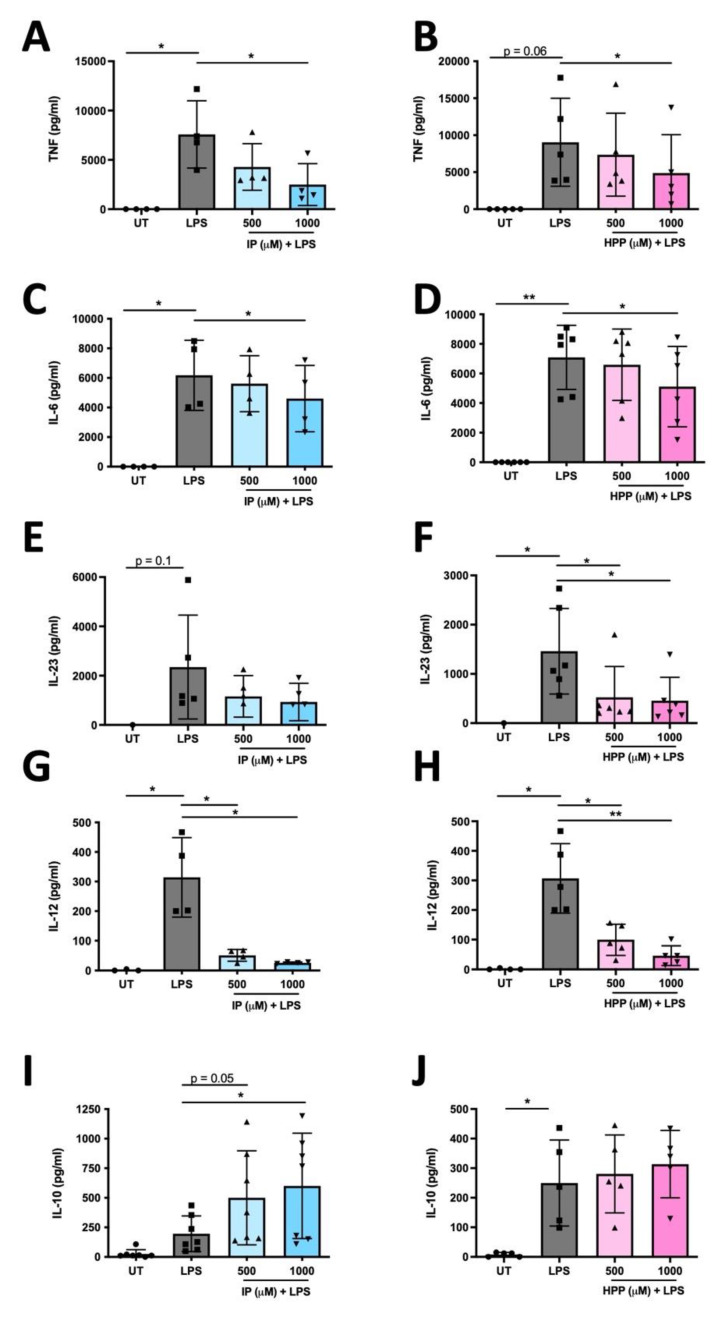
HPP and IP reduce the production of pro-inflammatory cytokines in LPS-stimulated human DC. Primary human DC were left untreated (UT) or incubated with IP (**A**,**C**,**E**,**G**,**I**) or HPP (**B**,**D**,**F**,**H**,**J**) (500–1000 µM) for 6 h prior to stimulation with LPS (100 ng/mL) for 24 h. Cell supernatants were assessed for TNF, IL-6, IL-23, IL-12p70, and IL-10 secretion by ELISA. Pooled data depict mean (±SEM) cytokine concentrations for four to seven healthy donors (means of three technical replicates per donor). Repeated measures one-way ANOVA, with Dunnett’s multiple comparisons post hoc test, was used to determine statistical significance, by comparing means of treatment groups against the mean of the control group (** *p* < 0.01, * *p* < 0.05).

We recently demonstrated that HPP and IP are capable of reducing the production of pro-inflammatory cytokines in murine glia and macrophages [5]. We next sought to determine if they have similar immune modulating activity in human DC. To test this, DC were treated with either HPP or IP (500–1000 μM) for 6 h prior to stimulation with LPS (100 ng/mL) for 24 h. Cytokine concentrations were measured in cell supernatants by ELISA. Both HPP and IP treatment dose-dependently reduced production of the pro-inflammatory cytokines TNF, IL-6, IL-12p70, and IL-23, and this effect was most potent at the 1000 μM concentration (Figure 3A–H). As well as driving the production of pro-inflammatory cytokines, LPS treatment over time is usually accompanied by production of the anti-inflammatory cytokine IL-10 as a means of regulating inflammatory responses. Interestingly, IP treatment resulted in a significant enhancement of IL-10, while HPP treatment showed a similar, albeit non-significant, trend (Figure 3I,J). These results suggest that both IP and HPP are capable of reducing the production of pro-inflammatory cytokines in LPS-stimulated DC, whilst also promoting a more anti-inflammatory phenotype.

### 3.4. HPP Treatment Inhibits the Maturation of LPS-Stimulated Human DC, Resulting in Reduced Activation of CD4^+^ T Cells

**Figure 4:**
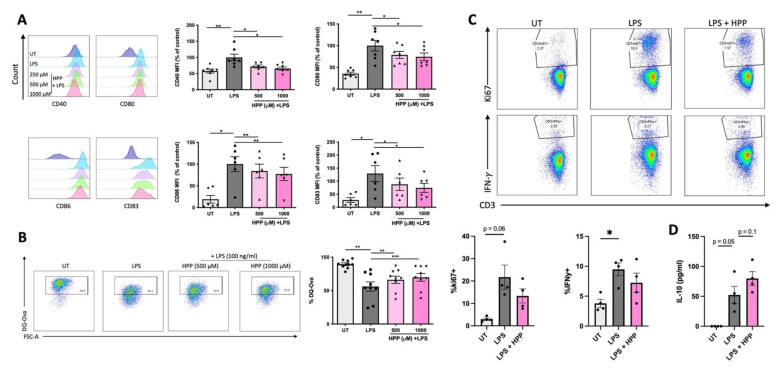
HPP treatment reduces DC maturation and subsequent CD4^+^ T cell activation. Primary human DC were left untreated (UT) or incubated with HPP (500–1000 μM) for 6 h prior to stimulation with LPS (100 ng/mL) for 24 h. (**A**) Cells were stained for CD40, CD80, CD86, and CD83 and analysed by flow cytometry. Histograms showing the expression of maturation markers for HPP-treated, LPS-stimulated DC compared to unstimulated cells or LPS stimulation alone from one representative experiment. Pooled data showing the mean (±SEM) MFI for each marker expressed as a percentage of control (LPS stimulation alone) from six to seven healthy donors. (**B**) DC were incubated with FITC-conjugated DQ-Ovalbumin (DQ-Ova; 500 ng/mL) for 20 min and were immediately acquired by flow cytometry. Dot plots depicting DQ-Ova uptake from one representative experiment. Pooled data showing the mean (±SEM) DQ-Ova uptake as a percentage of total cells from nine healthy donors. (**C**) DC were pre-treated with HPP prior to stimulation with LPS, and subsequently cultured with CD4^+^ T cells for five days. Dot plots depicting ki67 expression (as a measure of proliferation) and IFNγ expression from one representative experiment. Pooled data showing the mean (±SEM) of ki67^+^ and IFNγ^+^ cells as a percentage of CD3^+^CD8^−^ cells from four healthy donors. (**D**) Cell supernatants were assessed for IL-10 secretion by ELISA. Pooled data depict mean (±SEM) cytokine concentrations for four healthy donors (means of three technical replicates per donor). Repeated measures one-way ANOVA, with Dunnett’s multiple comparisons post hoc test, was used to determine statistical significance by comparing means of treatment groups against the mean of the control group (*** *p* < 0.001, ** *p* < 0.01, * *p* < 0.05).

In order to determine if the ketoacids impact DC maturation (and in turn T cell activation), human DC were treated with HPP (500–1000 μM) for 6 h prior to stimulation with LPS (100 ng/mL) for 24 h. Surface expression of maturation and co-stimulatory markers (CD40, CD80, CD83 and CD86) were measured by flow cytometry (due to the fluorescent nature of IP it is unsuitable for this flow cytometric analysis and hence was not included in these experiments). As expected, the expression of co-stimulatory markers was increased in LPS-stimulated DC. The average Median Fluorescent Intensity (MFI) of these cells was set to 100% and used as a control for comparison to the HPP-treated cells. There was a significant decrease in MFI at both HPP concentrations when compared to the LPS-stimulated control (Figure 4A). The phagocytic capacity of DC was next measured upon incubation of DC with FITC-conjugated DQ-Ovalbumin (DQ-Ova; 500 ng/mL) and uptake of the model antigen was assessed by flow cytometry. Compared to untreated cells, the ability of LPS-treated DC to phagocytose DQ-OVA was significantly impaired, signifying a heightened maturation status, however, pre-incubation with HPP prior to LPS treatment attenuated this effect and maintained the DC in an immature state (Figure 4B). Finally, and in order to determine if the reduced DC activation status that occurs in the presence of HPP has an impact on T cell activation, DC were treated with HPP (1000 μM) for 6 h prior to stimulation with LPS (100 ng/mL) for 24 h. The cells were then incubated with CD4^+^ T cells at a ratio of 10:1 (CD4^+^ T cells:DC) for five days. The supernatants were removed for cytokine analysis by ELISA and cells were stimulated with PMA (50 ng/mL), ionomycin (500 ng/mL) and brefeldin A (5 µg/mL) for 4 h. Expression of ki67, which is a measure of cell proliferation, was assessed by flow cytometry in CD3^+^CD8^−^ cells (gating strategy shown in Supplementary Figure S4), as was production of the pathogenic Th1 cytokine, IFNγ. T cells co-cultured with HPP-treated, LPS-stimulated DC showed a trend towards reduced ki67 and IFNγ expression when compared to T cells co-cultured with LPS-stimulated DC alone (Figure 4C). Interestingly, the T cells exhibited a trend towards enhanced production of the anti-inflammatory cytokine IL-10, when compared to T cells co-cultured with LPS-stimulated DC alone (Figure 4D). Overall, these results indicate that HPP reduces the maturation of innate DC, which in turn impacts adaptive T cell responses and may skew them towards a more anti-inflammatory phenotype.

### 3.5. HPP and IP Modulate Metabolic Reprogramming in LPS-Stimulated Human DC

**Figure 5:**
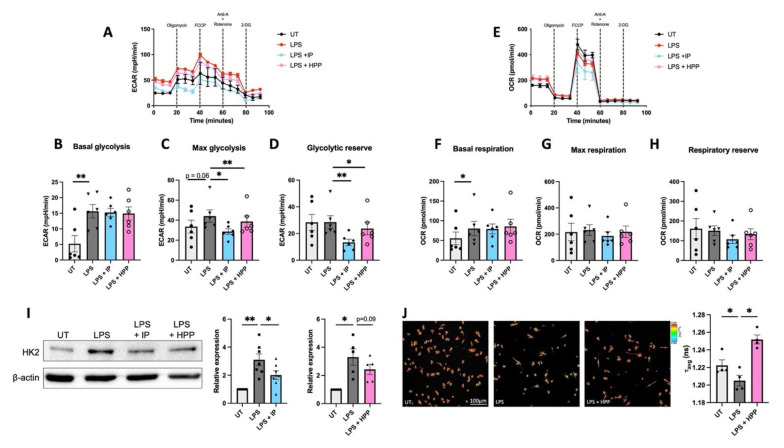
HPP and IP modulate metabolic reprogramming in LPS-stimulated DC. Primary human DC were pre-treated with either HPP or IP at 1000 μM for 6 h before stimulation with LPS (100 ng/mL) for 12 h. The extracellular acidification rate (ECAR) and the oxygen consumption rate (OCR) were measured using a Seahorse XFe96 analyser before and after the injections of oligomycin (1 mM), FCCP (1 mM), antimycin A (500 nM) and rotenone (500 nM), and 2-DG (25 mM). Bioenergetic profiles from one representative experiment depicting (**A**) ECAR and (**E**) OCR measurements over time. Pooled data (*N* = 6) depicts the calculated mean (±SEM) of (**B**) basal glycolytic rate, (**C**) max glycolytic rate, (**D**) glycolytic reserve, (**F**) basal respiratory rate, (**G**) max respiratory rate, and (**H**) respiratory reserve for each treatment group. (**I**) HK2 expression was measured by western blot. Densitometry results shown are mean ± SEM of the relative expression of HK2: β-actin from five to seven healthy donors. (**J**) FLIM images of DC measuring intracellular NADH. Pooled data (*N* = 4) depicts the mean (±SEM) of the ratio of bound:free NADH, represented by the *τ* average. Repeated measures one-way ANOVA, with Dunnett’s multiple comparisons post hoc test, was used to determine statistical significance by comparing means of treatment groups against the mean of the control group (** *p* < 0.01, * *p* < 0.05). ImageLab (Bio-Rad) software was used to perform densitometric analysis.

Metabolic reprogramming has been observed in immune cells and numerous recent studies have demonstrated that their activation/maturation is accompanied by a metabolic switch favouring glycolysis over oxidative phosphorylation [21]. In order to determine if *T. brucei*–derived ketoacids have an effect on immune cell metabolism, DC were pre-treated with IP or HPP (1000 μM) for 6 h prior to stimulation with LPS (100 ng/mL) for 12 h. These cells were then analysed in a Seahorse XFe96 analyser following the addition of oligomycin (1 mM), an inhibitor of mitochondrial complex V, FCCP (1 mM), a mitochondrial uncoupler, rotenone (500 nM) and antimycin A (500 nM), which are inhibitors of the mitochondrial complexes I & III, respectively, and 2-DG (25 mM), an inhibitor of glycolysis. The metabolic activity of the cells was then determined by measuring the ECAR, which is a measure of glycolysis, and the OCR, which is a measure of oxidative phosphorylation. IP- and HPP-treated DC showed no change in basal glycolysis when compared to LPS stimulation alone (Figure 5B). LPS-treated cells showed a trend towards increased max glycolysis, and this was significantly decreased in the presence of either HPP or IP (Figure 5C). Both IP and HPP were also capable of significantly decreasing the glycolytic reserve in LPS-stimulated cells (Figure 5C). There were no significant changes in the basal respiration (Figure 5F), max respiration (Figure 5G), and respiratory reserve (Figure 5H) in IP- or HPP-treated DC when compared to LPS stimulation alone, suggesting that they have no impact on oxidative phosphorylation.

Expression of HK2, the rate limiting enzyme in the glycolytic pathway, was next assessed by western blotting. HK2 is known to be induced by inflammatory stimuli and, as expected, LPS stimulation induced the upregulation of HK2 in DC. However, there was reduced expression of the enzyme in IP-treated DC, and a trend towards reduced expression (albeit not significant) in HPP-treated DC when compared to LPS-stimulated DC (Figure 5I). The effects of HPP treatment on the metabolism of DC was further investigated using FLIM, which measures the intracellular levels of NADH. Bound NADH, which is associated with oxidative phosphorylation, or free NADH, which is associated with glycolysis, can be distinguished based on their distinct lifetimes upon fluorescence excitation. The ratio of bound to free NADH can be used to measure whether a cell is favouring the engagement of glycolysis (a decrease in the ratio due to increased free NADH) or oxidative phosphorylation (an increase in the ratio due to increased bound NADH). Similar to the Seahorse results reported above, LPS-stimulated DC ramped up glycolysis, as represented by a decrease in the *τ* average compared to untreated DC (Figure 5J). Cells pre-treated with HPP exhibited a significant increase in the *τ* average compared to the LPS-stimulated controls, indicating they are favouring oxidative phosphorylation to generate their energy (Figure 5J). Overall, these results indicate that *T. brucei*-derived ketoacids modulate DC metabolism, reducing engagement of glycolysis, which is associated with rapid inflammatory responses.

### 3.6. HPP and IP Activate Autophagy-Related Proteins

**Figure 6:**
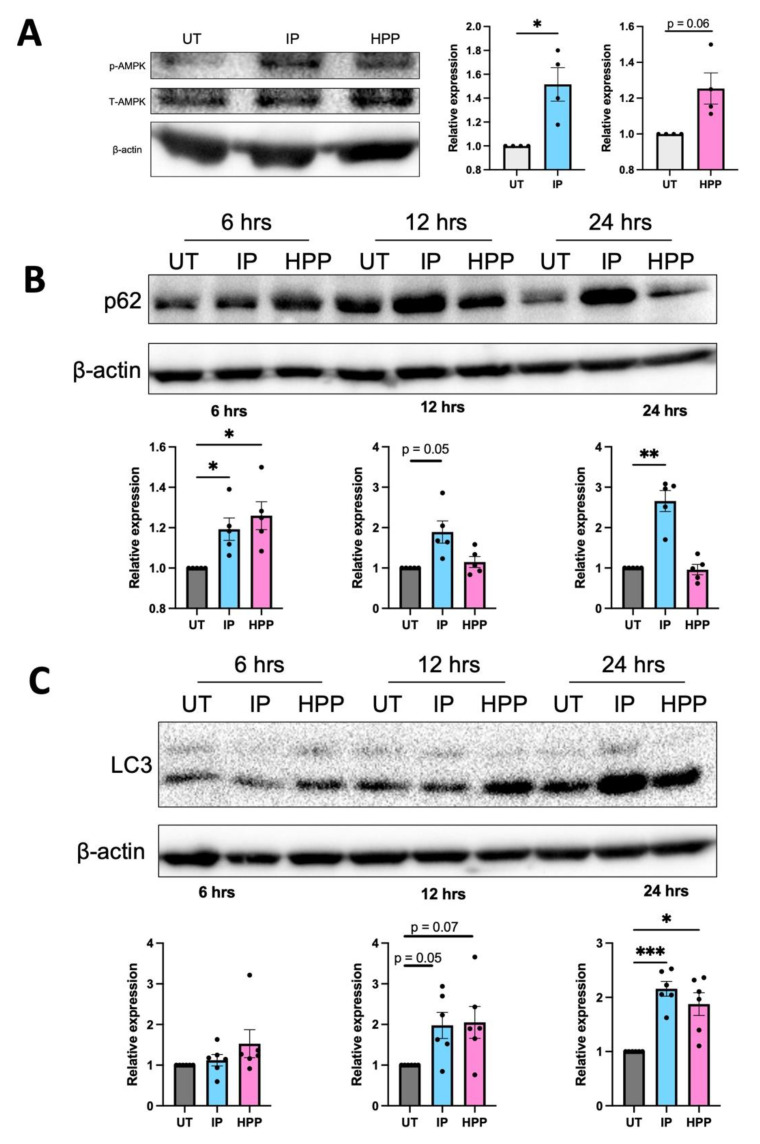
HPP and IP modulate autophagy-related proteins. (**A**) Primary human DC were left untreated (UT) or incubated with IP or HPP at 1000 µM for 15 min. Phosphorylation of AMPK was measured by western blot. Densitometry results shown are mean ± SEM of the relative expression of p-AMPK: β-actin from four healthy donors. (**B**,**C**) Primary human DC were left UT or incubated with IP or HPP at 1000 µM for 6, 12, or 24 h. Expression of (**B**) p62 and (**C**) LC3 were measured by western blot. Densitometry results shown are mean ± SEM of the relative expression of (**B**) p62: β-actin from five healthy donors and (**C**) LC3 II: β-actin from six healthy donors. (**A**) Statistical significance was determined using a Paired *t*-test. (**B**,**C**) Statistical significance was determined by repeated measures one-way ANOVA with Dunnett’s multiple comparisons post hoc test to compare means of treatment groups to the control group (*** *p* < 0.001, ** *p* < 0.01, * *p* < 0.05). ImageLab (Bio-Rad) software was used to perform densitometric analysis.

We have previously demonstrated that the HO-1 inducers carnosol and curcumin activate the autophagy regulator AMPK, which incidentally is also known to downmodulate glycolysis in immune activated cells [22]. Furthermore, the key autophagy-related protein, p62, is linked to Nrf2 activation [23,24,25]. In order to determine if *T. brucei*-derived ketoacids have any impact on autophagy-related proteins, DC were treated with IP or HPP (both 1000 µM) for 15 min and phosphorylation (and therefore activation) of AMPK was assessed by western blotting. IP treatment resulted in a significant increase in AMPK phosphorylation while there was a trend towards increased activation with HPP (Figure 6A). DC treated with IP also showed an increase in both p62 and LC3-II (which is converted from LC3-I during autophagy) over time, and this was most potent after 24 h (Figure 6B,C). HPP treatment significantly increased p62 expression after 6 h and significantly increased LC3-II expression after 24 h (Figure 6B,C). These results indicate both ketoacids are activating autophagy-related proteins in human DC.

### 3.7. HPP and IP Reduce Proliferation and Cytokine Expression in PBMC Isolated from IBD Patients

**Figure 7:**
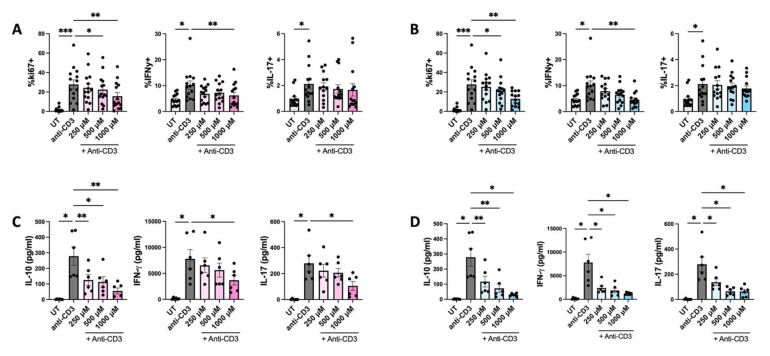
HPP and IP reduce proliferation and cytokine expression in ex vivo stimulated PBMC from patients with Inflammatory Bowel Disease. PBMC isolated from IBD patients were treated with (**A**,**C**) HPP or (**B**,**D**) IP (250 µM–1000 µM) for 6 h prior to stimulation with anti-CD3 for 12 h. After 18 h, culture media was replaced with fresh media and cells were incubated for a further 4 days with anti-CD3 stimulation. Supernatants were removed for analysis of cytokine concentration by ELISA. (**A**,**B**) Proliferation and cytokine production by CD3^+^CD8^−^ cells was analysed by flow cytometry. Pooled data (*N* = 14) depicting the mean ± SEM of ki67 (as a measure of proliferation), IFNγ, and IL-17 in CD3^+^CD8^−^ T cells. (**C**,**D**) Cell supernatants were assessed for concentrations of IL-10, IFNγ, and IL-17 by ELISA. Pooled data depicts mean (±SEM) cytokine concentrations for six IBD patients (means of three technical replicates per donor). Statistical significance was determined by repeated measures one-way ANOVA with Dunnett’s multiple comparisons post hoc test to compare means of treatment groups to the control group (*** *p* < 0.001, ** *p* < 0.01, * *p* < 0.05).

It has previously been reported that IP has powerful immune suppressive effects in a murine experimental colitis model [7]. In order to determine if these results translate to a more clinical setting, PBMC were isolated from IBD patients and treated with IP or HPP (250–1000 μM) for 6 h prior to stimulation with anti-CD3 (1 μg/mL) for a further four days. Furthermore, the culture media was replaced with fresh media after 18 h of incubation with the compounds to circumvent issues surrounding the fluorescence of IP over long periods of time. The supernatants were removed for cytokine analysis by ELISA and cells were stimulated with PMA (50 ng/mL), ionomycin (500 ng/mL), and brefeldin A (5 μg/mL) for 4 h. Expression of ki67, and the cytokines IFNγ and IL-17 (both of which are known to play a pathogenic role in IBD), were measured by flow cytometry in CD3^+^CD8^−^ cells (gating strategy shown in Supplementary Figure S5). IP was non-toxic to PBMC at all concentrations tested, however there was some toxicity seen when using the higher concentrations of HPP (Supplementary Figure S6). Despite this being significant, it is unlikely to account for the effects seen, as flow markers were examined in live cells only. Both IP- and HPP-treated cells were capable of dose-dependently reducing the proliferation and expression of IFNγ when compared to anti-CD3 stimulation alone (Figure 7A,B), while having no effect on the intracellular levels of IL-17. However, when cell supernatants were assessed by ELISA, there was a significant reduction in both IFNγ and IL-17 production in IP- and HPP-treated cells (Figure 7C,D). Unlike purified DC, the ketoacids did not enhance the production of IL-10 from PBMC, suggesting a cell-type specific effect (Figure 7C,D).

## 4. Discussion

The production of large amounts of immunomodulatory aromatic ketoacids during *T. brucei* infection likely serves to benefit the parasite by prolonging infection, proliferation, and, ultimately, survival in the host. While the secretome of *T. brucei* has been shown to reduce the secretion of IL-12, IL-10, IL-6, and TNF in both murine and human DC [26,27], the ketoacids, IP and HPP, have been shown to directly ameliorate inflammatory cytokine production in murine macrophages and glia [4,5]. This is further supported by studies demonstrating their therapeutic efficacy in murine models of disease [4,6,7]. Given the key role played by DC in shaping both innate and adaptive immune cell responses, we sought to determine if these effects translate to this vital human immune cell population, which not only serves to present antigens during infection, but also plays a key role in determining pathogenic T cell responses during disease. We demonstrate that both IP and HPP are capable of significantly reducing the secretion of a number of pro-inflammatory cytokines, including TNF, IL-6, IL-12, and IL-23 in LPS-stimulated DC. Furthermore, the ketoacids upregulate HO-1 in an Nrf2 dependant manner, which is in line with studies demonstrating that HO-1 induction can promote a more tolerogenic DC phenotype [18,19,28]. In support of this, we also demonstrate that ketoacid-treated DC have a reduced capacity to activate T cells, which in turn limits the production of the pathogenic T cell cytokine IFNγ, which is known to play a deleterious role in a number of inflammatory/autoimmune conditions including IBD [29].

In comparison to the previous data from murine macrophages and glia, a larger repertoire of cytokines is inhibited by the ketoacids in human DC. For example, IP and HPP had no effect on TNF secretion in murine glia and no effect on either TNF or IL-6 secretion in bone marrow derived macrophages [4,5], however, these cytokines were significantly reduced in LPS-activated human DC. Furthermore, IP directly induced the production of the anti-inflammatory cytokine IL-10 in DC. The maturation status of DC is also impacted by ketoacid treatment, with the HPP-treated cells exhibiting a reduction in co-stimulatory and maturation markers, which, in turn, prevents their ability to participate in T cell activation. The upregulation of HO-1 was also accompanied by the expression of additional Nrf2-regulated genes and both IP and HPP appeared to exhibit direct antioxidant activity, which may explain their ability to activate Nrf2, given its well documented capacity to rapidly respond to oxidative stress.

Further analysis of these novel HO-1 inducers also revealed that they can modulate immune cell metabolism. Indeed, recent studies have highlighted an important link between immune cell activation and metabolism. It is now well recognised that, not only do different immune cells engage different metabolic pathways, but that the activation/maturation state of the immune cells is accompanied by metabolic switches. For example, innate immune cells, including macrophages and DC, ramp up glycolysis in order to rapidly generate sufficient energy and the building blocks required to fight infection [21]. This phenomenon is also a feature of pathogenic immune cells, and a significant effort is underway to determine if controlling/preventing dysregulated metabolic reprogramming can serve to ameliorate detrimental immune cell activation during disease. Here we demonstrate that both IP and HPP can decrease the max glycolysis observed in LPS-treated DC while also downregulating the expression of hexokinase 2, the rate-limiting enzyme in glycolysis. These results are similar to our recent observations with the HO-1 inducers, carnosol and curcumin, suggesting that HO-1 may be an important regulator of immune cell metabolism [30]. Further study is required to determine if additional features of metabolism are affected by ketoacids and whether metabolic reprogramming occurs during *T. brucei* infection, but these in vitro results are in line with the notion that metabolism is intricately linked with immune cell activation, and that the downmodulation of glycolysis in immune cells promotes a more tolerogenic phenotype.

From a therapeutic stance, IP in particular has shown potential in a murine model of colitis where administration of the ketoacid not only improved disease outcome, but also decreased expression of pro-inflammatory cytokines, including IL-12, IFNγ, and TNF [7]. We observed similar results in PBMC from IBD patients where both HPP and IP reduced the proliferation of anti-CD3 stimulated T cells, as well as the secretion of both IL-17 and IFNγ. Indeed, induction of HO-1 is being explored as a therapy for IBD and has shown promise in a number of murine models of disease [31,32,33,34,35]. Patients are currently treated largely with anti-inflammatories including 5-aminosalicylic acid (5-ASA), corticosteroids, methotrexate, and anti-TNF therapies [36]. However, many patients are/become refractory to these treatments and will require surgery in their lifetime. While further in vivo study is required to fully elucidate their efficacy and provide further information regarding treatment route, dosing, and long-term effects, these findings provide further impetus to explore aromatic ketoacids as a treatment for IBD (and indeed other inflammatory diseases), either alone or in combination with existing therapies.

Finally, a particularly interesting finding of this study is the observation that IP and HPP can activate key autophagic proteins. Autophagy itself is carried out by a number of autophagy-related (Atg) proteins and initiation is under the control of the protein kinases mTOR and AMPK, which are both intrinsically linked to immune cell metabolism (AMPK inhibits glycolysis while mTOR activation is linked to induction of glycolysis). The autophagic process is complicated and involves many different proteins and has been reviewed in detail elsewhere [37,38,39]. Briefly, a complex of Atg proteins lipidates LC3-I converting it to LC3-II [37,38]. LC3-II binds to the autophagosome membrane and facilitates the docking of cargos and proteins for degradation through their binding to p62 [40]. Following maturation, the autophagosome fuses with the lysosome to form the autolysosome, the contents of which are then degraded and recycled. In this study, we show that IP and HPP activate AMPK and increase expression of p62 and LC3-II. Autophagy is of particular importance for DC, as many of their key functions, including antigen uptake and presentation, are strongly associated with autophagy [41]. Despite some conflicting reports, generally it appears that activation of autophagy gives rise to a more tolerogenic DC phenotype, exhibiting reduced antigen presentation and maturation [41], which is similar to the results seen in this study. Furthermore, AMPK activation has been shown to attenuate pro-inflammatory cytokine production and DC maturation [22,42]. Therefore, ketoacid-induced AMPK and subsequent autophagy activation may serve to downmodulate DC maturation, antigen presentation, and glycolysis.

Notably, p62 is not only an important autophagy-related protein, it also plays a crucial role in the activation of Nrf2 [23,24,25]. Nrf2 is activated upon release from KEAP1, which can occur when p62 sequesters KEAP1, targeting it for degradation and allowing Nrf2 to translocate to the nucleus [23,24,25]. Therefore, the activation of p62 by both ketoacids, but in particular IP, may be responsible for the subsequent activation of Nrf2, and therefore HO-1, by these ketoacids. While HPP induces HO-1 at an earlier time than IP, the latter appears to have more potent effects overall. In most cases, the in vitro effects of IP are most apparent at 24 h, and we cannot rule out the possibility that it is converting to another form over time. Further study is undoubtedly required to delineate the true impact of aromatic ketoacids (and their potential derivatives) and HO-1 induction on autophagy-related processes, in addition to the noted effects on DC metabolic reprogramming.

## 5. Conclusions

In conclusion, the data presented here expands our understanding of the mechanism of action of *T. brucei*-derived ketoacids in human immune cells and suggests that HO-1 induction may be useful to regulate the metabolism and, therefore, function of immune cells in inflammatory disease. We firmly believe that these compounds represent novel and exciting HO-1 inducers worthy of further exploration.

## Figures and Tables

**Table 1:** Primer sequences. Table containing the forward and reverse primer sequences for NQO1, GSR, and β-actin.

Gene	Forward Primer	Reverse Primer
NQO1	5′ TGAAGAAGAAAGGATGGGAG 3′	5′ TTTACCTGTGATGTCCTTTC 3′
GSR	5′ GACCTATTCAACGAGCTTTAC 3′	5′ CAACCACCTTTTCTTCCTTG 3′
β-actin	5′ GGACTTCGAGCAAGAGATGG 3′	5′ AGCACTGTGTTGGCGTACAG 3′

## Data Availability

The data is contained within the article or supplementary material.

## Supplementary Materials

The following supporting information can be downloaded at: https://www.mdpi.com/article/10.3390/antiox11010164/s1, Figure S1: HPP and IP are non-toxic to human DC; Figure S2: IP and HPP are not contaminated with endotoxin; Figure S3: ML385 is non-toxic to human DC; Figure S4: Gating strategy used to generate data shown in Figure 4; Figure S5: Gating strategy used to generate data shown in Figure 6; Figure S6: IP is non-toxic to PBMC, while higher concentrations of HPP shows significant, albeit mild, reductions in viability; Table S1: Seahorse calculations.
