# Supplementary material for: The Trypanosoma brucei-Derived Ketoacids, Indole Pyruvate and Hydroxyphenylpyruvate, Induce HO-1 Expression and Suppress Inflammatory Responses in Human Dendritic Cells
Source: Antioxidants (Basel). 2022 Jan 15;11(1):164. doi: 10.3390/antiox11010164 (PMC8772738; doi:10.3390/antiox11010164)
Supplement: Supplementary file 1 [file antioxidants-11-00164-s001.zip › antioxidants-1541092-SI.pdf]

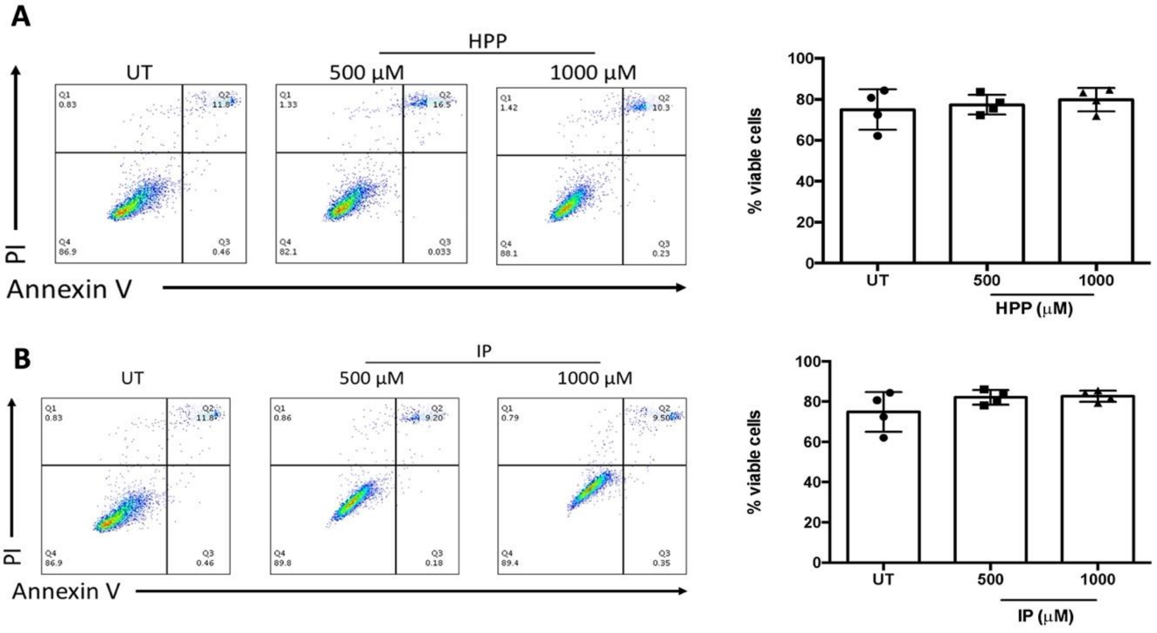

Supplemental Figure S1. HPP and IP are non-toxic to human DC. Primary human DC were left untreated (UT) or incubated with **(A)** HPP or **(B)** IP (500 and 1000 µM) for 24 hours. Cells were stained for Annexin V and PI uptake, as indicators of apoptosis, and analysed by flow cytometry. Debris was excluded by gating, and viable cells were defined as Annexin V$^-$ PI$^-$ (non-apoptotic cells). Dot plots depicting Annexin V and PI expression from one representative experiment. Pooled data showing the mean (± SEM) of Annexin V$^-$ PI$^-$ cells as a percentage from 4 healthy donors.

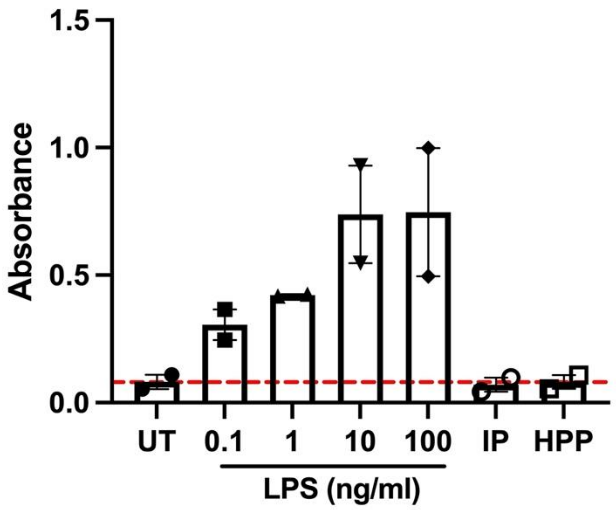

Supplementary Figure S2. IP and HPP are not contaminated with endotoxin. HEK-blue cells expressing TLR4 were left untreated (UT), stimulated with LPS (0.1–100 ng/ml; positive control), or incubated with HPP or IP (both 1000 µM) for 24 hours. The expression of SEAP which is under the control of NF-kB and AP-1 was tested by incubating cell supernatants with HEK-blue detection medium for 30 min at 37 °C and absorbance was read at 650 nm. Pooled data showing the mean (± SEM) absorbance readings from two independent experiments.

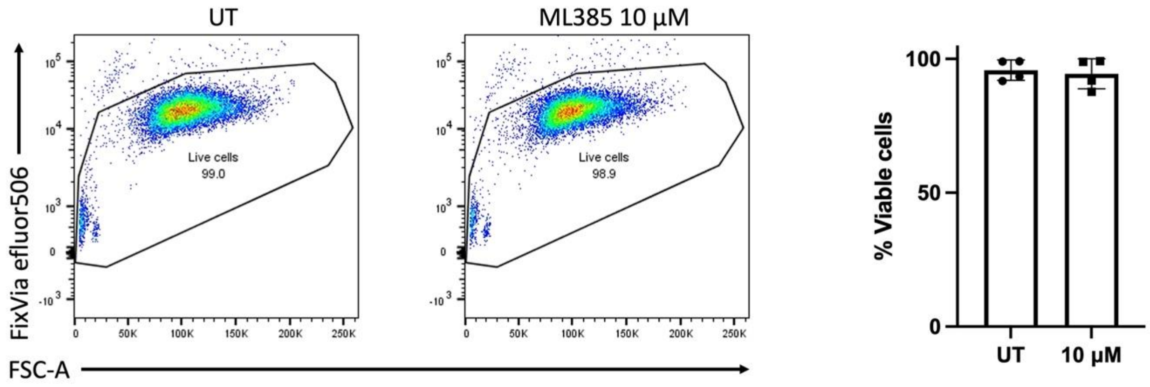

Supplemental Figure S3. ML385 is non-toxic to human DC. Primary human DC were left untreated (UT) or incubated with ML385 (10 μM) for 24 hours. Cells were stained for viability using FixVia efluor506. Debris was excluded by gating and viable cells were defined as FixVia efluor506^{-}. Dot plots depicting FixVia efluor506 expression from one representative experiment. Pooled data showing the mean (± SEM) of viable cells as a percentage from 4 healthy donors.

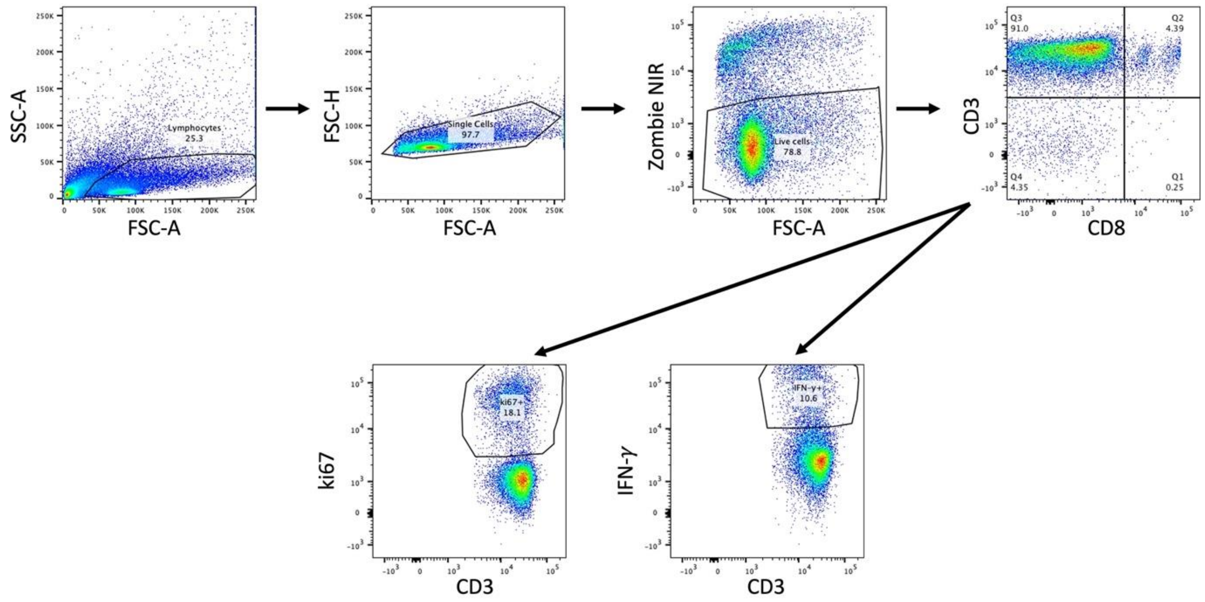

Supplemental Figure S4. Gating strategy used to generate data shown in Figure 4. To assess proliferation and cytokine production in T cells co-cultured with DC, lymphocytes were gated on based on forward and side scatter and doublets were excluded. Viable single cells were then gated on by excluding cells which had taken up the viability dye. The CD4 T cell population was approximated by gating on $CD3^{+}CD8^{-}$ cells (Q1), as CD4 is often downregulated during restimulation with PMA and ionomycin. Representative dot plots for IFNγ and ki67 expression in the $CD3^{+}CD8^{-}$ population are shown. Cytokine gates were set using unstimulated PBMC incubated in the presence of brefeldin A.

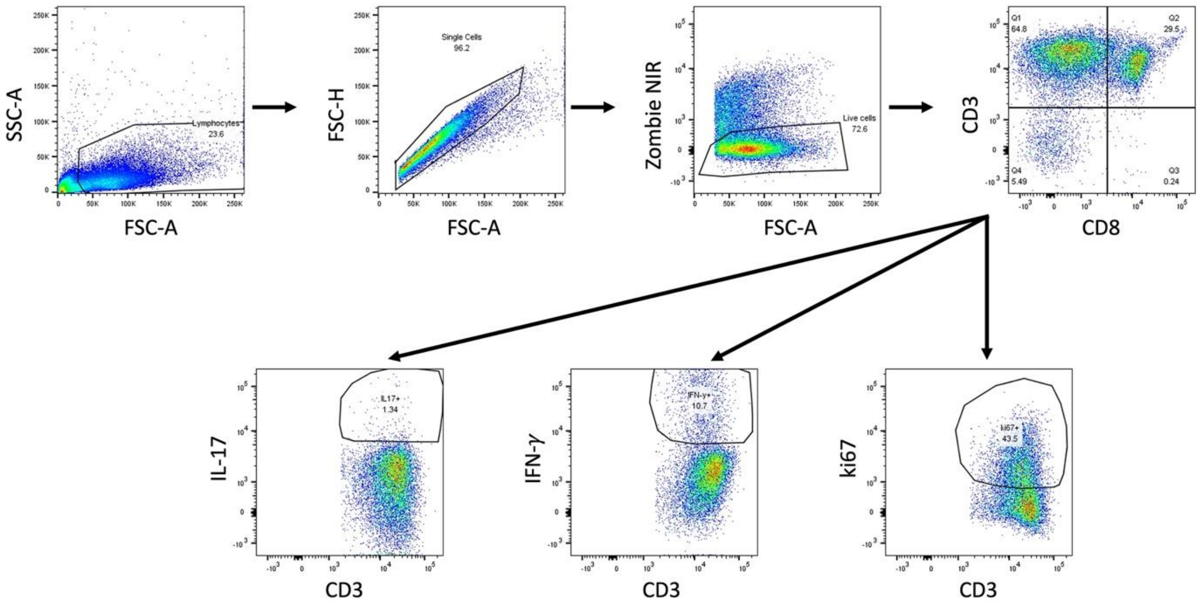

Supplemental Figure S5. Gating strategy used to generate data shown in Figure 6. To assess proliferation and cytokine production in ex-vivo stimulated PBMC from patients with inflammatory bowel disease, lymphocytes were first gated on based on forward and side scatter and doublets were excluded. Viable cells were then gated on by excluding cells which had taken up the viability dye. The CD4 T cell population was approximated by gating on $CD3^{+}CD8^{-}$ cells (Q1), as CD4 is often downregulated during restimulation with PMA and ionomycin. Representative dot plots for IFNγ and ki67 expression in the $CD3^{+}CD8^{-}$ population are shown. Cytokine gates were set using unstimulated PBMC incubated in the presence of brefeldin A.

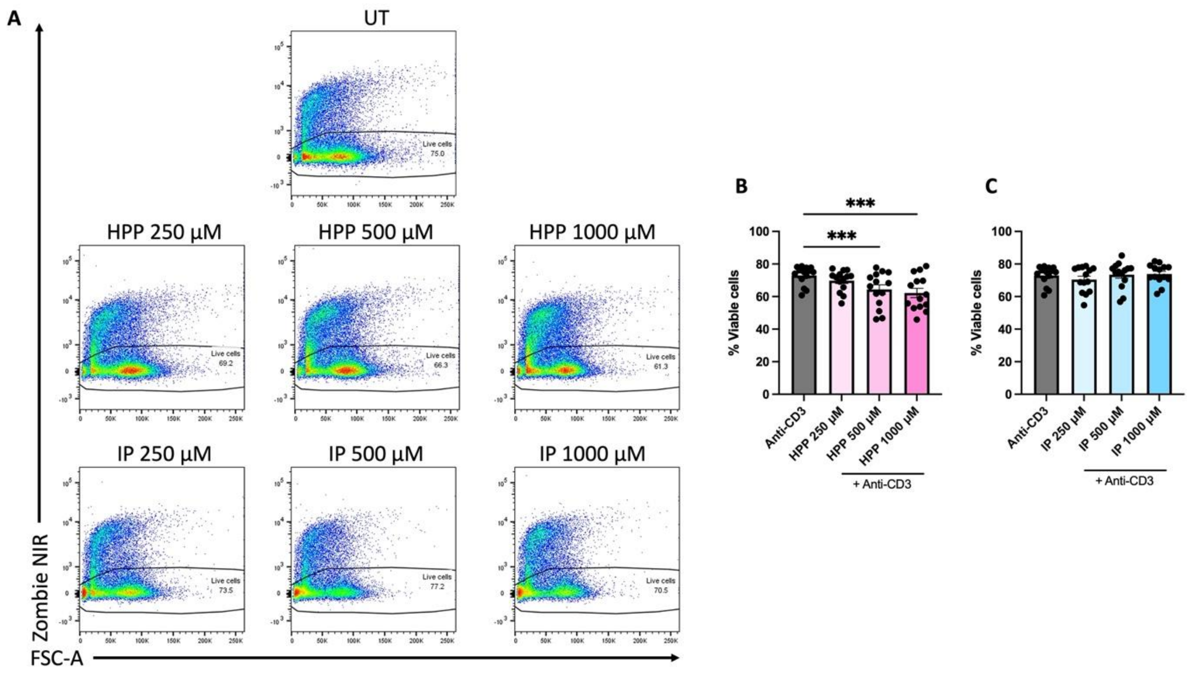

Supplemental Figure S6. IP is non-toxic to PBMC, while higher concentrations of HPP shows significant, albeit mild, reductions in viability. PBMC isolated from IBD patients were treated with **(B)** HPP or **(C)** IP (250 µM – 1000 µM) for 6 hours prior to stimulation with anti-CD3 for 12 hours. The media was replaced with fresh media, and incubated for a further 4 days with anti-CD3 stimulation. Cells were stained for viability using Zombie NIR^{TM} Fixable Viability kit. Debris was excluded by gating, and viable cells were defined as Zombie NIR^{-} . **(A)** Dot plots depicting Zombie NIR expression from one representative experiment. **(B&C)** Pooled data showing the mean (± SEM) of viable cells as a percentage from 14 IBD patients.

Rate	Calculation
Basal glycolysis	Average ECAR values prior to oligomycin treatment – non-glycolytic ECAR
Max glycolysis	Average ECAR values after oligomycin and before FCCP
Glycolytic reserve	Max glycolysis – basal glycolysis
Basal respiration	Average OCR values prior to oligomycin treatment – non-mitochondrial OCR
Max respiration	Average OCR values after FCCP & before Rotenone/antimycin A treatment
Respiratory reserve	Max respiration – basal respiration

Supplementary Table S1. Seahorse calculations. Table outlining the calculations used to determine the measurements for the Seahorse assay in Figure 5.
